# Changes in Health Care Expenditure after the Loss of a Spouse: Data on 6,487 Older Widows and Widowers in the Netherlands

**DOI:** 10.1371/journal.pone.0115478

**Published:** 2014-12-23

**Authors:** Herbert J. A. Rolden, David van Bodegom, Rudi G. J. Westendorp

**Affiliations:** 1 Leyden Academy on Vitality and Ageing, Leiden, The Netherlands; 2 Department of Gerontology and Geriatrics, Leiden University Medical Center, Leiden, The Netherlands; Iran University of Medical Sciences, Iran, Republic of Islamic

## Abstract

**Background:**

In ageing populations, informal care holds great potential to limit rising health care expenditure. The majority of informal care is delivered by spouses. The loss of informal care due to the death of the spouse could therefore increase expenditure levels for formal care.

**Objective:**

To investigate the impact of the death of the spouse on health care expenditure by older people through time. Additionally, to examine whether the impact differs between socio-demographic groups, and what health services are affected most.

**Design:**

Longitudinal data on health care expenditure (from July 2007 through 2010) from a regional Dutch health care insurer was matched with data on marital status (2004–2011) from the Central Bureau of Statistics. Linear mixed models with log transformed health care expenditure, generalized linear models and two-part models were used to retrieve standardized levels of monthly health care expenditure of 6,487 older widowed subjects in the 42 months before and after the loss of the spouse.

**Results:**

Mean monthly health care expenditure in married subjects was €502 in the 42 months before the death of the spouse, and expenditure levels rose by €239 (48%) in the 42 months after the death of the spouse. The increase in expenditure after the death of the spouse was highest for men (€319; 59%) and the oldest old (€553; 82%). Expenditure levels showed the highest increase for hospital and home care services (together €166).

**Conclusions:**

The loss of the spouse is associated with an increase in health care expenditure. The relatively high rise in long-term care expenses suggests that the loss of informal care is an important determinant of this rise.

## Introduction

In many developed countries, population ageing can be directly related to a rise in health care expenditure. In the Netherlands, for example, the share of people aged 65 and above is expected to increase from 15% in 2008 to 26% in 2040 [1] and health care expenses increase exponentially after the age of 65 [2]. Although higher levels of health care expenditure are associated with old age, older people also hold potential to limit expenditure levels. By offering informal care, older people keep their partners, family-members, friends or neighbours away from the health care sector. Not much known is about the economic value of this preventive role of the older population.

It is plausible that informal care from spouse to spouse accounts for the biggest share expenditure prevention. It has been found that women offer the highest share of informal care, either as wives or daughters [3]. The contribution of men rises with age, and relates mainly to spouses [4]. Several authors have found that having a partner significantly decreases older people’s risk of utilizing nursing home services [5]–[9]. However, these studies do not include expenditure levels and often do not look at the use of other health care services. The economic value of spousal informal care can be estimated by observing the difference in health care expenditure by people with a spouse with that of people who have no spouse, or no longer have a spouse. It has been found that the death of a spouse increases subsequent mortality risk [10]–[12], health risks [13], risk of long-term care utilization [14], [15], and medical care utilization [16]. In a study of 61 widows and widowers by Prigerson, Maciejewski, & Rosenheck (2000) it was estimated that widowed persons in the United States incurred up to 60% more health care expenditure than married persons [17]. Besides the observation in this small study, the effect of the death of the spouse on health care expenditure through time remains largely unknown.

Therefore, in a large and complete dataset of Dutch health care expenditure, we have investigated the impact of the death of the spouse on health care expenditure through time. We collected figures of individual health care expenditure within a large population over a long period of time from the database of a health care insurer, and linked this information with data on marital status. First, we analysed the effect of having a spouse and other socio-demographic determinants on health care expenditure in our total study population. Second, we focused only on clients who became widowed, and observed if individual levels of health care expenditure change before and after the death of the spouse. We also discerned whether there is a difference in this effect between men and women, younger and older people, and people with a lower and higher socio-economic status. Third, we investigated how expenditure levels for different health services change before and after the death of the spouse. Insight into the economic value of formal care prevention by spouses is important for policy-makers who are concerned with improving the lives of older people and who are, at the same time, obliged to curtail ever rising levels of health care expenditure.

## Methods

### Ethics statement

After consulting the internal review board (IRB) of the regional health insurer, data on health care expenditure was retrieved from a health insurer. A formal waiver of IRB approval was received from the Central Bureau of Statistics (CBS) for data collection. After consulting the IRB of the CBS a single transfer of data from the health insurer to the Central Bureau of Statistics (CBS) was undertaken over a secure line. A formal waiver was received by the IRB of the CBS. After the data transfer, the CBS first removed any personal data. The leading author could then only access the de-identified data in a secured room of the CBS. The authors had no access to identifying information. Any output destined for publication was first scrutinized by the IRB of the CBS, so no output could be traced back to individuals. No data is publicly available. Data collection and analysis was in full accordance with privacy legislation and protocol.

### The Dutch health care system

Health care in the Netherlands can be separated into two main sectors: medical care and long-term care. Medical care refers to consultations, medication and treatment from general practitioners, medical specialists, dentists, pharmacists, and therapists (such as physiotherapists and psychotherapists). Some forms of instrumental aid and transportation are also provided through the medical care sector. In the studied time period, health care providers bill health care insurers in the form of diagnosis related groups. Medical care is legally arranged through the Health Insurance Act (HIA).

Long-term care in the Netherlands is legally arranged through the Exceptional Medical Expenses Act (EMEA). Entitled to care through the EMEA are people who cannot satisfy their basic care needs independently due to a physical, psychogeriatric or psychiatric ailment, or a mental, physical or sensorial handicap. Before someone may receive long-term care through the EMEA, the Center for Indication Setting has to evaluate the client’s health status and issue an official indication. When an indication is set for a client, the actual provision of long-term care is arranged by so-called *care offices.* After an indication is set, the care office appoints a health care provider for a client, or distributes a personal budget. The health care insurer who has the highest share of clients in a region acts as the care office for that region.

There were basically five separate long-term care services funded through the EMEA in the studied time period: (1) personal care (e.g. dressing, undressing, help with bathing or showering), (2) nursing care (e.g. wound dressing), (3) counseling (daytime activities and help with improving self-reliance, individually tailored or in groups), (4) treatment (forms of rehabilitation or therapy) and (5) residence. Indications for extramural care are defined in type and level (hours per week). Since July 1^st^ 2007, intramural care is indicated in terms of Care Weight Packages (CWPs). CWPs are pre-defined bundles of residential care, consisting of housing with different types of long-term care on different levels.

### Data

With the aim to perform multiple studies on the association between the life situation of older people and their health care costs, the *Leiden Health care Costs in Old Age* (LHCOA) study was started in 2011. For this study, data on health care expenditure for 61,495 people aged 65 and older in a period of 42 months were retrieved from a regional Dutch health insurer (*Zorg & Zekerheid*) and matched with data on socio-economic characteristics from the Central Bureau of Statistics (CBS).

Data were collected in the following steps:

1. After consulting the internal review board (IRB) of the regional health insurer, data on medical care expenditure were retrieved from the health insurer’s management information system. Data were collected for the period July 2007 through 2010 for all persons who lived in the regions where the health care insurer acted as the long-term care office, and who reached the age of 65 before 2011. Addresses were linked with data on socio-economic status by postal code, provided by the Netherlands Institute for Social Research.
2. Collecting data on expenditure for separate medical care services proved to be highly time consuming. These data were only retrieved for a subpopulation, randomized for socio-economic status. Eight separate services were identified: general practice, hospital, pharmacy, allied health care, psychology, instrumental aids, dental care, and other medical care.
3. In accordance with the IRB of the health insurer, data on long-term care utilization were collected from the EMEA Care Registration system (ECR), an information system which offers an oversight of all the coded messages that are sent between organizations active within the confounds of the EMEA. ECR messages designating the start and end of long-term care provision were linked with national average fees per volume unit, provided by the Health Insurance Board. The received volumes of extramural treatment were unknown, and no expenses could be calculated for this service.
4. After consulting the IRB of the CBS a single transfer of data from the health insurer to the CBS was done over a secure line. CBS staff merged the data using citizen service numbers and dismissed any personal data afterwards. The authors could then only access the de-identified data in a secured room of the CBS. The authors had no access to identifying information. Any output destined for publication was first scrutinized by the IRB of the CBS, so no output could be traced back to individuals. No data is publicly available. Data collection and analysis was in full accordance with privacy legislation and protocol. Socio-demographic variables collected at the CBS were: age, gender, marital status, and time of death.
5. After these steps, the total study population comprises 61,495 subjects. Expenditure for separate medical care services are known for 18,995 subjects.

**Figure 1:**
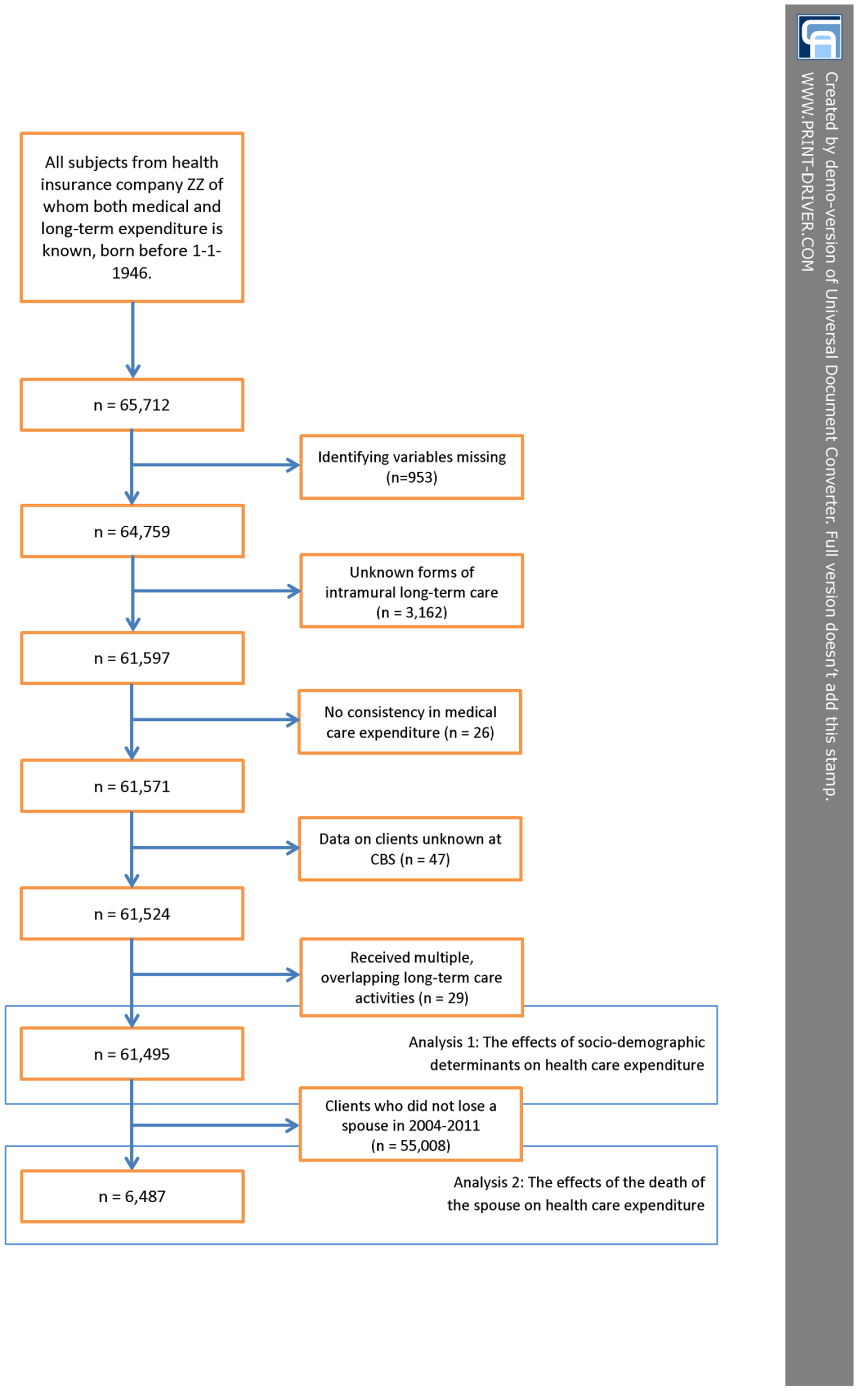
Flowchart of data collection.

A more detailed description of the data collection procedure can be found in Fig. 1, which presents a flowchart of the procedure.

### Statistical analysis

In the overall analysis, we used the total study population (n = 61,495) to investigate what influence gender, age, marital status, socio-economic status (SES), and calendar year have on monthly health care expenditure (HCE). All determinants are defined as categorical variables. We have defined two groups for age (65–79 years old, and 80 years and older) and SES (score <6.45 and score ≥6.45) based on the medians (age = 77 years; SES score = 6.44). As there are multiple, monthly observations for each subject in our dataset, and HCE is skewed to the right, leptokurtic and heteroskedastic, we used a linear mixed model with a log transformation of HCE (LLMM). Visual inspection of the distribution of the residuals in the model now showed a normal distribution. The model can be expressed with the following equation:
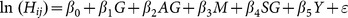
(1)where *H* stands for HCE for individual *i* in month *j*, *G* for gender, *AG* for age group, *M* for marital status, *SG* for socio-demographic status group, and *Y* for calendar year. To estimate the effect of each determinant in the equation, we calculated the exponential function of each *β* in the equation.

Hereafter, the focus was shifted to the subjects who became widowed in the period 2004–2011 (n = 6,487). Data on marital status was available for a longer time period (2004–2011) than data on HCE (July 2007–2010). This way, we could measure the impact of the death of the spouse on HCE with a maximum time span of 42 months before and 42 months after the month in which the subjects became widowed. A LLMM was used to estimate HCE for every month. Standardization was applied for socio-demographic variables and calendar year by holding them constant on the mean. Monthly point estimates of HCE before and after the loss of the spouse were also calculated for different socio-demographic groups: both genders, two age groups, and two socio-economic status groups. The LLMM used in the second analysis can be expressed with the following equation:
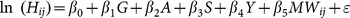
(2)where *H* stands for HCE for individual *i* in month *j* (*j* =  42 to +42), *G* stands for gender, *A* for age, *S* for socio-demographic status score, *Y* for calendar year, and *MW* for each month before or after widowhood.

Monthly points estimates will provide eye evidence on a possible effect of widowhood on HCE. To test whether the level of HCE during widowhood differs significantly from the period before widowhood, we compared the mean monthly levels of both periods in a two-sample t-test. The means of HCE for each period were calculated from the estimates from the LLMM (equation 2).

In analysing the effect of the loss of the spouse on expenditure levels in specific health services, we selected subjects for whom expenditure were known for each separate medical care service and who became widowed from 2004 to 2011 (n = 2,027). For some health services, only a relative low number of subjects received care. As the outcome variables were thus not only characterized by problems with skewness, kurtosis and heteroskedasticity, but also with a high share of zero values, a LLMM could not be applied. Therefore, we first pooled monthly expenses of each health service for every subject, and then used either a generalized linear model (GLM) or two-part model (2PM) with a logit model for the first part, and a GLM with gamma distribution and a log link for the second part [18], [19]. The GLM can be expressed as:
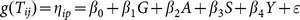
(3)where *g* is the link function, and *T* stands for average monthly expenditure for a specific health service for individual *i* in period *p*, *G* stands for gender, *A* for average age, *S* for socio-demographic status, and *Y* stands for average calendar year. The 2PM is expressed as:



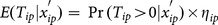
(4)where *x′* expresses the set of determinants, and *η* the GLM (see equation 3 for *x′* and *η*). For each health service, comparison of the average monthly expenditure levels before the loss of the spouse with the average level after the death of the spouse was performed with a two-sample t-test.

## Results

### The study populations

The descriptive statistics of the total study population, the widowed population and the widowed subpopulation can be found in table 1. First, we analysed the effects of socio-demographic characteristics on health care expenditure (HCE) in the total population (n = 61,495). Hereafter, we included those subjects who lost their spouse in the period 2004–2011 to investigate the impact of the death of the spouse on HCE (n = 6,487). The majority of these widowed subjects are women (71%). The average follow-up before the death of the spouse is 11.6 (±14.6) months, and after the death of the spouse 16.8 (±12.8) months, both with a maximum of 42 months. For the analysis concerning expenditure for different health services, a widowed subpopulation was used (n = 2,027). Characteristics of the widowed subpopulation are similar to the total widowed population.

**Table 1:** Characteristics of the study populations.

	Total population		Widowed population		Widowed subpopulation	
	*Number (%)*		*Number (%)*		*Number (%)*	
**Study population**	61,495		6,487		2,027	
**Gender**						
Men	24,904	(40%)	1,854	(29%)	590	(29%)
Women	36,591	(60%)	4,633	(71%)	1,437	(71%)
**Age**						
65–79	39,113	(64%)	4,729	(73%)	1,442	(71%)
≥80	12,010	(20%)	1,758	(27%)	585	(29%)
**Marital status**						
Married	35,291	(57%)	3,139	(48%)	991	(49%)
Widowed	16,819	(27%)	3,348	(52%)	1,036	(51%)
Divorced	5,742	(9%)	–	–	–	–
Never married	3,643	(6%)	–	–	–	–
**Socio-economic status (SES)**						
Lower (SES score <6.45)	30,633	(50%)	3,288	(51%)	1,232	(61%)
Higher (SES score ≥6.45)	30,862	(50%)	3,199	(49%)	795	(39%)

Descriptive statistics are all baseline figures.

### Effects of socio-demographic characteristics on health care expenditure

Table 2 shows the impact socio-demographic determinants have on HCE in the total study population (n = 61,495). In the total population, mean monthly health care expenditure is €629. The level of HCE for widows and widowers is €329 higher than for married people, which equals 52% of the mean. HCE for divorced subjects is €49 (8%) higher, and for those never married HCE is €196 (31%) higher compared to married subjects. On average, women have €20 (3%) more HCE per month than men, and subjects of 80 years or older have €316 (50%) more HCE than their younger counterparts. Expenditure levels are €25 (4%) lower for subjects with a higher socio-economic status than for subjects with a higher socio-economic status.

**Table 2:** The effect of socio-demographic characteristics on health care expenditure (HCE) in the total study population (n = 61,495), from July 2007 through 2010.

	Effect size*	
	(€/month)	p
**Gender**		
Men	Ref	
Women	+20	.002
**Age**		
65–79	Ref	
80+	+316	<.001
**Marital status**		
Married	Ref	
Widowed	+329	<.001
Divorced	+49	<.001
Never married	+196	<.001
**Socio-economic status (SES)**		
Lower SES group	Ref	
Higher SES group	–25	<.001

Average monthly HCE of the study population is 629 euro.

*Results are coefficients and their p-value from a linear mixed model with log transformation.

### The effect of the death of the spouse on health care expenditure

**Figure 2:**
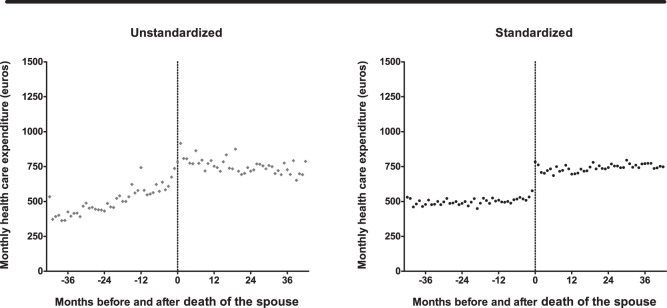
Monthly health care expenditure (HCE) before and after death of the spouse (n = 6,487). Point estimates in the left panel represent the raw data. Point estimates from the right panel are from a linear mixed model with log transformed HCE, standardized for gender, age, socio-economic status, and calendar year.

In the second analysis, we only focus on the subjects who became widowed in the period 2004–2011. As can be seen from Fig. 2 (left panel), HCE rises gradually before the loss of the spouse and shows a steep increase in four months around the month the spouse died. The month in which a subject became widowed is defined as “month 0”, and depicted as a vertical dotted line. After the initial rise in HCE after the death of the spouse, there is a slight decline in HCE. As these results are unstandardized, results could be influenced by a rise in the mean age, changing shares of men vs. women and lower vs. higher socio-economic classes, as well as rising health care prices. For this reason, we regressed the monthly HCE on the months before and after the death of their spouse in a linear mixed model with log transformation. Fig. 2 (right panel) shows the monthly point estimates from the regression model, standardized for age, gender, socio-economic status score, and calendar year. With standardization, HCE shows no more gradual rise before the loss of the spouse, and no slight decline afterwards. There is a sudden increase in HCE of €207 (36%) in the month the subjects lose their spouse.

**Figure 3:**
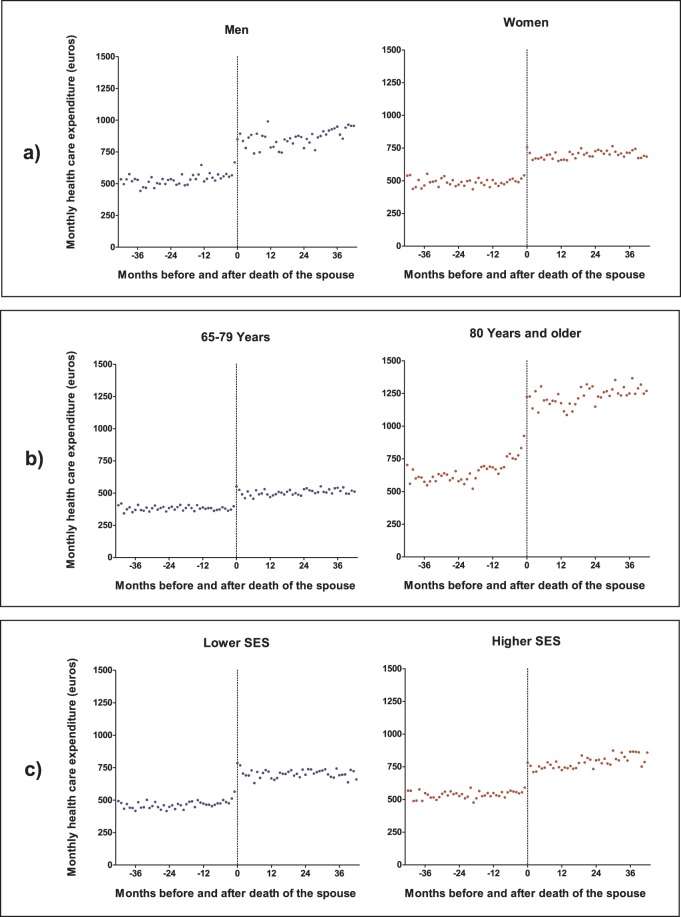
Monthly health care expenditure before and after the death of the spouse for both genders (a), two age groups (b) and two SES groups (c). Point estimates are from a linear mixed model with log transformed health care expenditure, standardized for gender, age, socio-economic status and calendar year.

Fig. 3 shows the monthly points estimates of HCE in the months before and after the loss of the spouse for both genders (panel a), two age groups (b) and two SES groups (c). The expenditure level of men rises to a higher level after the death of the spouse than for women. After the loss of the spouse, men have a continual increase in HCE, while the level of women remains more stable. Individuals in the older age group have higher levels of HCE than their younger counterparts before and during widowhood. An increase in monthly HCE starts to set in earlier for the older age group (around 7 months before death of the spouse) than for the younger age group, for which no real increase in HCE is noticeable prior to widowhood. Widowed subjects in the lower SES group require slightly less HCE before and after the death of the spouse. Individuals of a higher socio-economic class have a small, continual rise in HCE after the death of the spouse.

The mean levels of HCE in the 42 months before and after the loss of the spouse in the widowed population, and different socio-demographic groups, are shown in table 3. Overall, HCE is €239 higher in the 42 months after the death of the spouse than in the 42 months before, which is equal to a rise of 48% (p = 0.01). With €319 (59%; p = 0.116) the rise in HCE is higher for men than for women (€209; 43%; p = 0.042). When still married, individuals in the older age group have higher levels of HCE (€678) than their younger counterparts (€381). This difference further increases after the death of the spouse: HCE for the older age group rises with €553 (82%; p = 0.028) and for the younger age group with €124 (32%; p = 0.12). The level of HCE for individuals from the higher socio-economic group is higher before and after the death of the spouse, but the relative increase due to widowhood is lower (45% vs. 50%). However, this rise in HCE after the death of the spouse is not significant for both socio-economic groups.

**Table 3:** Differences in health care expenditure (HCE) in the 42 months before and after death of the spouse (population of widowed subjects, n = 6,487).

	Mean monthly HCE (€)		Difference		
	Before death of the spouse	After death of the spouse	€	%	p
**All**	**502**	**741**	**239**	**48**	**.010**
**Gender**					
Men	540	859	319	59	.116
Women	489	698	209	43	.042
**Age**					
65–79	381	505	124	32	.120
80+	678	1231	553	82	.028
**Socio-economic status**					
Lower SES group	469	703	234	50	.056
Higher SES group	539	784	245	45	.077

Mean monthly levels of HCE are from a linear mixed model with log transformed HCE, standardized for gender, age, socio-economic status, and calendar year (see Figs. 2 and 3).

### The effect of the death of the spouse on expenditure for separate health services

In table 4, the association between HCE and widowhood over time is shown for each health service in a subpopulation of 2,027 widows and widowers. With €271 (56%;p<0.001) the overall effect size of the death of the spouse on HCE is similar to the total widowed population. The rise in the absolute level of expenditure levels after the death of the spouse is slightly larger in the medical care (€140; p<0.001) sector than in the long-term care sector (€131; p<0.001). However, the relative rise is five times smaller in the medical care sector than in the long-term care sector (30% versus 157%). Expenditure for nursing home care shows the highest relative increase after the death of the spouse with 163%, although this increase is not significant (p = 0.057). Expenditure for hospital services increases the most in the absolute sense (€110 per month; p<0.001). The lowest absolute increase takes place in the field of general practice (€4; p<0.001).

**Table 4:** Differences in health care expenditure (HCE) in the 42 months before and after death of the spouse for separate health services, standardized for gender, age, socio-economic status and calendar year (subpopulation of widowed subjects, n = 2,027).

	Mean monthly HCE (€)		Difference		
	Before death of the spouse	After death of the spouse	€	%	p
**Total** a	**486**	**757**	**271**	**56**	**<.001**
**Medical care** a	**379**	**519**	**140**	**30**	**<.001**
General practice^a^	18	22	4	23	<.001
Hospital^b^	196	306	110	56	<.001
Pharmacy^a^	78	84	6	8	.219
Other^1^^,^ b	87	106	20	23	.288
**Long-term care** b	**107**	**238**	**131**	**157**	**<.001**
Home care^b^	55	111	56	103	<.001
Counselling^b^	12	27	15	120	.017
Care home^b^	11	24	14	132	.051
Nursing home^b^	16	43	27	163	.057
Other^2^^,^ b	13	32	19	144	.013

a Results are from a generalized linear model (GLM).

b Results are from a two-part model with logit model in the first part, and GLM in the second.

1 Dental care, allied health care, mental health care, transportation, instrumental aids, acupuncture, and others.

2 Long-term rehabilitation, palliative care, care for the handicapped, and long-term mental health care.

## Discussion

The death of the spouse impacts the level of health care expenditure of an older person. On average, monthly expenditure increases with almost 50% after the death of the spouse. Overall, the rise sets in at the month someone becomes widowed, and is highest for men and the oldest old. The relative increase in long-term care expenditure after the loss of the spouse is five times higher in the long-term care sector than in the medical care sector.

### Marriage protection, marriage selection, a shared environment, bereavement

Since spouses can provide each other with intensive levels of informal care, one could argue that marriage has preventive effects on formal care use. Although the loss of a spouse is treated as an exogenous shock, different endogenous factors could be at play in the association between widowhood and health care expenditure. One could argue from a marriage selection perspective [20]. People tend to select their spouse on the basis of similar characteristics, such as age, intelligence, educational level, and psychological well-being [21], [22]. Because spousal partners are similar in these traits, it is likely that their health care expenditure levels also show similar patterns over time, regardless of life events. Another plausible explanation is that married couples share a similar environment and are thus susceptible to equal risk factors [23]. Both effects cause widowed people to make more use of health services, not because of the death of the spouse, but because they have a higher chance of being ill, just as their partner was. We accounted for these endogenous factors to the best of our ability by standardizing for socio-demographic factors.

Bereavement is also related to health risks, and could therefore induce higher health care expenditure [24], [25]. The point estimates portrayed in Figs. 2 and 3 suggest that bereavement plays a minor role in the association between widowhood and health care expenditure as there is no peak in expenditure in the months after the death of the spouse. Also, the increase in health care expenditure after the death of the spouse is five times higher in the long-term care sector than the medical care sector. This suggests that expenditure levels predominantly rise because there is a higher need for formal care after the loss of an informal care-giver, rather than a higher need for medical treatment related to the health impact of bereavement, marriage selection or a shared environment.

Other studies take the effects of marriage selection, a shared environment, and bereavement into account by directly measuring the “substitution” effect of informal care reception on health care utilization [26]–[31]. These authors show that informal care has a preventive role, or in other words, that care provided by spouses, family members, and friends substitutes for formal health care. It is plausible that this substitution effect is a major contributing factor to the association we found between widowhood and health care expenditure. Because our data are retrieved from a follow-up study, we can separate the direct effects of bereavement, and by standardizing for age and socio-economic status we have at least partly avoided the interference of marriage selection and a shared environment.

### The economic value of informal care and prevention programs

Our findings are particularly useful for economists studying the impact of human behaviour or the costs and benefits of prevention programs. Because informal care is not financially compensated, it is usually not taken into account in economic analyses. In literature, it remains unclear what the best method is to express informal care in economic terms [32]. The economic value of formal care use prevention can be used as a method to value the dynamics of informal care as an alternative to, for example, the “proxy-good method” [33], or the “willingness-to-pay method” [34]. Research on the economic burden of smoking is an example of a field in which the economic valuation of informal care is lacking. Some authors have concluded that smoking benefits the economy, because smokers usually don’t reach higher, more expensive ages [35]–[37]. However, these studies focus only on individual health care expenditure during the life course and fail to take into account that people outliving their smoking partners have a higher chance of requiring formal care. A part of the higher average levels of life course health care expenditure of non-smokers is thus caused by smoking partners who cause second-hand smoke and die younger. Consequently, when the effect of second-hand smoke and informal care are taken into account in these analyses, the real costs of smoking are higher than currently reported.

This study can also benefit policy-makers and physicians. For example, results show that health care expenditure of older people is not only higher after the death of the spouse, but also in the months before widowhood. Emotional stress and a higher burden of informal care could be an underlying dynamic. It is also possible that some subjects postponed the use of health care services for themselves until intensive care for their dying spouse was no longer required. Intervention programmes including information, support, therapy, and respite care for older people providing intensive care for their spouses could have financial merit besides any potential beneficial effects on well-being [38]. Similarly, widows and widowers may benefit from an expanded role for general practitioners. Older people can suffer from confusion, apathy, dependency, and depression after the death of their spouse. A pro-active general practitioner can assist and support those recently widowed to improve their independence and well-being, also diminishing the need for medical treatment or care in the long run [39]. Our results suggest there is room for such interventions, as the level of expenditure in the sector of general practice increases just slightly (€4 per month) after the death of the spouse.

An important strength of our paper is that individual characteristics and expenditure levels were collected within a large study population over a relatively long time period (July 2007 through 2010). However, we could only collect data on marital status and some widows and widowers will have started a new relationship outside the bonds of marriage. Also, information on the causes of death of the spouse, or the actual reception of informal care before widowhood was missing. Additionally, the effect of the death of the spouse on health care expenditure is likely mediated by the presence of children, as well as other family members or friends. On the other hand, not all widowed subjects from our study received informal care prior to widowhood, showing that informal care provided in spousal relations is economically more valuable than the extra 239 euros per month that widowed people cost. Hence, we want to emphasize that we have measured the effect of having a spouse on health care expenditure and not the effect of informal care per se.

To our knowledge, we are the first to perform an in depth investigation into the effect of the loss of a spouse on health care expenditure through time. Our results are in line with previous papers on the utilization of health care by widowed people. Our research not only points out the value of informal care to society in economic terms, but also provides further basis to develop interventions for the support of widows and widowers. This way, future financial strains on the health care system can be avoided, and, more importantly, the quality of life of widows and widowers can be improved.
